# Optimal partial regularity of very weak solutions to nonhomogeneous A-harmonic systems

**DOI:** 10.1186/s13660-017-1297-z

**Published:** 2017-01-18

**Authors:** Qing Zhao, Shuhong Chen

**Affiliations:** School of Mathematics and Statistics, Minnan Normal University, Fujian, Zhangzhou 363000 China

**Keywords:** nonhomogeneous A-harmonic systems, very weak solution, optimal partial regularity, Hodge decomposition, A-harmonic approximation technique

## Abstract

We study partial regularity of very weak solutions to some nonhomogeneous A-harmonic systems. To obtain the reverse Hölder inequality of the gradient of a very weak solution, we construct a suitable test function by Hodge decomposition. With the aid of Gehring’s lemma, we prove that these very weak solutions are weak solutions. Further, we show that these solutions are in fact optimal Hölder continuity based on A-harmonic approximation technique.

## Introduction

We consider optimal partial regularity of very weak solutions to nonhomogeneous A-harmonic systems of the following type:

1.1 $$ -\operatorname{div} A(x,u,Du)=f(x), $$

where $u:\Omega \rightarrow R^{N}$ is a vector-valued function on a bounded domain $\Omega \subset R^{n}$ ($n\geqslant 2$), and $Du=\{D_{ \alpha} u^{i}\}$ ($1\leqslant \alpha \leqslant n,1\leqslant i\leqslant N$) stands for the gradient matrix of *u*, $A(x,u,\xi ):\Omega \times R ^{N} \times {R^{nN}\rightarrow R^{nN}}$ is a measurable function, and $A_{i}^{\alpha} (x,u,\xi)$ ($1\leqslant \alpha \leqslant n,1\leqslant i\leqslant N$) are of class $C^{1}$ in *ξ*. To define the very weak solutions to systems (1.1) and obtain the optimal partial regularity results, we need to impose certain structural and regularity conditions on *A* and to restrict *u* and *f* to a particular class of functions as follows: for some $p\geqslant 2$,

1. *A* is a bounded operator, that is, there exists a constant $\beta >0$ such that
  $$\begin{aligned} \bigl\vert A(x,u,\xi ) \bigr\vert \leqslant \beta \bigl(1+\vert \xi \vert ^{2} \bigr)^{\frac{p-1}{ 2} } \quad \mbox{for all } (x,u,\xi )\in \Omega \times R^{N} \times R ^{nN}; \end{aligned}$$
2. *A* is differentiable with respect to $\xi \in R^{nN}$, that is, there exists a constant $\alpha >0$ such that
  $$\begin{aligned} D_{\xi} A(x,u,\xi )\zeta \cdot \zeta \geqslant \alpha \bigl(1+\vert \xi \vert ^{2} \bigr)^{ \frac{{p-2}}{2 }}\vert \zeta \vert ^{2} \end{aligned}$$
  for all $x\in \Omega ,u\in R^{N}$, and $\xi ,\zeta \in R^{nN}$;
3. there exist a constant $\gamma \in (0,1) $ and a nondecreasing function $K: [0,\infty )\rightarrow [0,\infty )$ such that
  $$\begin{aligned} \bigl\vert A(x,u,\xi )-A(\tilde{x},\tilde{u},\xi ) \bigr\vert \leqslant K \bigl(\vert u \vert \bigr) \bigl(\vert x-\tilde{x}\vert ^{p}+ \vert u-\tilde{u}\vert ^{p} \bigr)^{\frac{\gamma }{p}} \bigl(1+ \vert \xi \vert \bigr)^{\frac{p}{2}} \end{aligned}$$
  for all $x,\tilde{x} \in \Omega ,u,\tilde{u}\in R^{N}$ and $\xi \in R^{nN}$; without loss of generality, we take $K \geq 1 $;
4. *f* is a given vector field in $R^{N}$ of class $L_{\mathrm{loc}}^{\frac{nq}{n(p-1)+q}}(\Omega )$, $q>p$.

Under these assumptions, we can now define very weak solutions to (1.1).

### Definition 1

A mapping $u\in W_{\mathrm{loc}}^{1,r}(\Omega ),p-1\leqslant r< p$, is called a very weak solution to (1.1) if

1.2 $$ \int_{\Omega} A(x,u,Du)\cdot D\phi \,dx= \int_{\Omega} f(x) \phi \,dx $$

for all $\phi \in W_{0}^{1,\frac{r}{r-p+1}}(\Omega )$.

In order to improve the integrability of a very weak solution to (1.1), we need to prove a suitable reverse Hölder inequality. In 1973, Gehring [1] discovered the crucial self-improving property of the reverse Hölder inequality and applied it to establish higher integrability of *n*-dimensional *k*-quasiconformal mapping. Subsequently, Meyers and Elcrat [2] generalized this inequality based on Caccioppoli’s inequality. They improved the integrability of weak solutions to nonlinear elliptic systems with the help of Gehring’s lemma. Especially, they pointed out that regularity properties remained valid in a somewhat slightly larger Sobolev space to linear elliptic systems depending on the duality. In fact, this regularity result about very weak solutions was first showed by Meyers [3] in 1963. Unfortunately, neither the method used in [2] for proving the reverse Hölder inequality nor the duality employed in [2, 3] can be applied to deal with very weak solutions to nonlinear elliptic systems. To overcome these difficulties, Lewis [4] used the technique of harmonic analysis and successfully proved that very weak solutions to nonlinear elliptic systems are indeed weak solutions. Later Iwaniec and Sbordone [5] achieved a similar result via the methods of Hodge decomposition and prior estimation.

Since then, studies on properties of very weak solutions to partial differential equations, especially for regularity of very weak solutions to A-harmonic systems, have attracted considerable attention. Following the method of Iwaniec and Sbordone [5], Giachetti, Leonetti, and Schiachi [6] obtained the partial regularity result of A-harmonic systems $\operatorname{div} A (x,u,Du)=0$. Tong, Gu, and Xu [7] extended their result to nonhomogeneous A-harmonic systems $\operatorname{div} A(x,Du)=f(x)$ and improved the integrability of very weak solutions. Greco, Iwaniec, and Sbordone [8] even applied this method to the *p*-harmonic equation $\operatorname{div} \vert Du \vert ^{p-2}Du=\operatorname{div} f$.

Motivated by these works, we mainly consider the optimal partial regularity to nonhomogeneous A-harmonic systems in the form of (1.1) under assumptions (H1)-(H4).

For the sake of desired results, we first need to improve the exponent of integrability for the gradient of a very weak solution to an even slightly better one than the natural exponent *p*. The crucial difficulty is to construct an appropriate test function below the natural exponent. In this article, we follow the spirit of Iwaniec and Sbordone [5] using the Hodge decomposition to construct it. Combining the Sobolev imbedding theorem, Young’s inequality, Poincaré’s inequality, and so on, we improve the exponents of integrability of very weak solutions to (1.1). In other words, we successfully prove that very weak solutions to (1.1) are in fact weak solutions. More precisely, we obtain the following result.

### Theorem 1

*Let* *u* *be a very weak solution to systems* (1.1). *Assume that the structure conditions* (H1), (H2), *and* (H4) *hold*. *Then there exist exponents* $p-1< r_{1}=r_{1}(n,N,p,\alpha ,\beta )< p< r_{2}=r_{2}(n,N,p, \alpha ,\beta )<\infty $ *such that* $u\in W_{\mathrm{loc}}^{1,r_{1}}(\Omega )$ *belongs to* $W_{\mathrm{loc}}^{1,r_{2}}(\Omega )$.

A direct consequence of this result follows immediately.

### Corollary 1

*Under the assumptions of Theorem* 1, *there exists* $r_{1}=r_{1}(n,N,p, \alpha ,\beta )< p$ *such that every very weak solution* $u\in W_{\mathrm{loc}} ^{1,r}(\Omega )$ *with* $r_{1}< r< p$ *belongs to* $W_{\mathrm{loc}}^{1,p}(\Omega )$.

Further, we establish the optimal partial regularity result of very weak solutions to (1.1). Generally speaking, we cannot expect that weak solutions to (1.1) will be $C^{2}$-solutions even under reasonable assumptions on operator *A* and *f*. This is initially pointed out by De Giorgi [9, 10] and Giusti and Miranda [11]. Thus, our aim is to obtain the optimal Hölder continuity of very weak solutions to (1.1). Fortunately, we achieve it by means of A-harmonic approximation technique and obtain the optimal Hölder continuity $C^{1,\gamma }$ in the regular set of the following:

### Theorem 2

*Let* $u\in W_{\mathrm{loc}}^{1,r}(\Omega )$, $r_{1}< r< p$, *be a very weak solution to* (1.1). *Consider* $r_{1}$ *as in Corollary* 1. *Suppose that assumptions* (H1)-(H4) *hold*. *Then there exists an open set* $\Omega_{0}\subset \Omega $ *such that* $u\in C^{1,\gamma }(\Omega_{0})$ *for* *γ* *is defined in* (H3). *We have*

$$\begin{aligned} \Omega -\Omega_{0}=\Sigma_{1}\cup \Sigma_{2}, \end{aligned}$$

*where*

$$\begin{aligned} \Sigma_{1}= \biggl\{ x_{0} \in \Omega : \hskip4pt \liminf _{R \to 0^{+}} \fint_{B_{R}(x _{0})} \bigl\vert Du-(D u)_{x_{0},R} \bigr\vert ^{p} \,dx >0 \biggr\} \end{aligned}$$

*and*

$$\begin{aligned} \Sigma_{2}= \Bigl\{ x_{0} \in \Omega : \hskip4pt \limsup _{R \to 0^{+}} \bigl(\vert u_{x_{0},R} \vert + \bigl\vert (D u)_{x_{0},R} \bigr\vert \bigr)= \infty \Bigr\} . \end{aligned}$$

*Moreover*, *we have* $\vert \Omega -\Omega_{0} \vert =0$.

To close this section, we briefly summarize the notation used in this paper. As noted before, we consider a bounded domain $\Omega \subset R ^{n}(n\geqslant 2)$ and mappings from Ω to $R^{N}$. We write $B_{r}(x_{0})=\{x\in \Omega : \vert x-x_{0} \vert < r\},x_{0}\in \Omega $. For a given set *X*, we denote by $\vert X \vert $ its *n*-dimensional Lebesgue measure. If $\vert X \vert >0$, then the average of a given $g\in L^{1}(X)$ over *X* is denoted by $\fint_{X} g \,dx$, that is, $\fint_{X} g \,dx=\frac{1}{\vert X \vert }\int_{X}g \,dx$. In particular, we write $g_{x_{0},r}=\fint_{B_{r}(x_{0})}g\,dx $. Let $\alpha_{n}$ denote the volume of the unit ball in $R^{n}$, that is, $\alpha_{n}=\vert B_{1}(0) \vert $, then $\vert B_{r}(x_{0}) \vert =\alpha_{n} r^{n}$.

The rest of this paper is arranged as follows. In Section 2, we provide some necessary preliminary lemmas. In Section 3, we prove the main results.

## Preliminary lemmas

Before proving the results, we state a few useful lemmas.

The first one is a stability result of the Hodge decomposition, from which we could construct a suitable test-function concerning estimates below the natural exponent for (1.1).

### Lemma 1

[5]

*Let* $\Omega \subset R^{n}$ *be a regular domain*, *and* $w\in W_{0} ^{1,r}(\Omega ,R^{N}),r>1$, *and let* $-1<\epsilon <r-1$. *Then there exist* $\phi \in W_{0}^{1, \frac{r}{1+\epsilon }}(\Omega ,R^{N})$ *and a divergence*-*free matrix field* $H\in L^{\frac{r}{1+\epsilon }}(\Omega ,R ^{nN})$ *such that*

2.1 $$ \vert D w \vert ^{\epsilon} D w=D\phi +H. $$

*Moreover*,

2.2 $$ \Vert H \Vert _{\frac{r}{1+\epsilon }}\leqslant C_{r}( \Omega , N)\vert \epsilon \vert \Vert D w \Vert _{r}^{1+\epsilon }. $$

The most useful case for us in Lemma 1 is where *ϵ* is negative. For $u\in W_{\mathrm{loc}}^{1,r}(\Omega )$ with $p-1< r< p$ that is a very weak solution to (1.1), we can set $\epsilon =r-p$ $(-1<\epsilon <0)$. Then there exists $\phi \in W_{0}^{1,\frac{r}{1+r-p}}(\Omega )$; thus, *ϕ* can be illustrated as a test-function in (1.2). In view of (2.1) and (2.2), we also can get an estimate of *Dϕ*, which is similar to (2.2).

Applying Lemma 2, we can decompose the left term of the Hodge decomposition into two terms that could be controlled more easily in the proof of Theorem 1.

### Lemma 2

[12]

*For every* $X, Y \in R^{n}$, $X\neq 0, Y\neq 0$, *and* $0\leqslant \epsilon <1$, *we have the inequality*

$$\begin{aligned} \bigl\vert \vert X \vert ^{-\epsilon }X- \bigr\vert Y \bigl\vert ^{-\epsilon }Y \bigr\vert \leq 2^{\epsilon} \frac{1+ \epsilon }{1-\epsilon }\vert X-Y \vert ^{1-\epsilon }. \end{aligned}$$

In the end of this section, we shall introduce a form of Gehring’s lemma, which plays an important role in the proof of Theorem 1. It implies in particular that from it higher integrability of $g(x)$ follows.

### Lemma 3

[2, 13]

*Let* $0< R< R_{0}\leqslant \operatorname{dist} (x_{0},\partial \Omega ),x _{0}\in \Omega $. *Suppose that* $g(x)\in L^{p} (B_{R}(x_{0}) )$, $f(x) \in L^{t} (B_{R}(x_{0}) ),t>p,1< p<\infty $, *satisfy the reverse Hölder inequality*

$$\begin{aligned} \fint_{B_{R/2}(x_{0})} \bigl\vert g(x) \bigr\vert ^{p}\,dx \leqslant \theta \fint_{B_{R}(x_{0})} \bigl\vert g(x) \bigr\vert ^{p}\,dx +C^{*} \biggl[ \fint_{B_{R}(x_{0})} \bigl\vert g(x) \bigr\vert ^{s}\,dx \biggr]^{p/s} + \fint_{B_{R}(x_{0})} \bigl\vert f(x) \bigr\vert ^{p}\,dx \end{aligned}$$

*for some* $1\leqslant s< p,0\leqslant \theta <1$. *Then* $g\in L_{\mathrm{loc}} ^{p'}(\Omega )$ *for some* $p'=p'(\theta ,p,n,C^{*})$ $(t\geqslant p' >p)$, *and*

$$\begin{aligned} \biggl[ \fint_{B_{R/2}(x_{0})} \bigl\vert g(x) \bigr\vert ^{p'}\,dx \biggr]^{1/{p'}} \leqslant C_{*} \biggl[ \fint_{B_{R}(x_{0})} \bigl\vert g(x) \bigr\vert ^{p}\,dx \biggr]^{1/p} +C_{*} \biggl[ \fint_{B_{R}(x_{0})} \bigl\vert f(x) \bigr\vert ^{p'}\,dx \biggr]^{1/{p'}}, \end{aligned}$$

*where* $C_{*}=C_{*}(n,C^{*},p,\theta ,R_{0})$.

## Proof of the main theorems

In this section, we give a proof of partial regularity results. Consider *u* solving (1.1) on $B_{R}(x_{0})\Subset \Omega $, where we restrict $0< R< R_{0}<\min\{1, \operatorname{dist}(x_{0},\partial \Omega )\}$.

### Proof of Theorem 1

#### Proof

Fix a cut-off function $\eta \in C_{0}^{\infty }(B_{R}(x _{0}))$ satisfying $0\leqslant \eta \leqslant 1$, $\vert D \eta \vert \leqslant C/R$, and $\eta \equiv 1$ on $B_{R/2}(x_{0})$. Let $u\in W_{\mathrm{loc}}^{1,r}(B _{R}(x_{0}))$ with $p-1< r< p$ be a very weak solution to (1.1). Denote $u-u_{x_{0},R}-p_{0}(x-x_{0})$ by *v*, where $p_{0}\in R^{nN}$. We find that *v* has calculus mean-value 0 on $B_{R}(x_{0})$, that is, $v_{x_{0},R}=0$. Notice that $\eta v \in W_{0}^{1,r}(B_{R}(x_{0}))$ and $-1< r-p<0$. Then there exist $\phi \in W_{0}^{1,\frac{r}{1+r-p}}(B _{R}(x_{0}))$ and $h\in L^{\frac{r }{{1+r-p}}}(B_{R}(x_{0}))$ such that $\vert D(\eta v) \vert ^{r-p}D(\eta v)=D \phi +h$ according to the Hodge decomposition. Thus, *ϕ* is admissible as a test-function in the definition of very weak solutions. Set $-\varepsilon =r-p$ ($-1<-\varepsilon <0$) for convenience. Then $r=p-\varepsilon $, and we have

3.1 $$ \bigl\vert D(\eta v) \bigr\vert ^{-\varepsilon }D(\eta v)=D \phi +h, $$

where *h* satisfies

3.2 $$ \Vert h \Vert _{\frac{p-\varepsilon }{1-\varepsilon }}\leqslant C_{r}( \Omega ,N) \varepsilon \bigl\Vert D (\eta v) \bigr\Vert _{p-\varepsilon }^{1-\varepsilon }. $$

Further, applying Poincaré’s inequality with constant $C_{P}$ and noting that $v_{x_{0},R}=0$, we get

3.3 $$\begin{aligned} \bigl\Vert D(\eta v) \bigr\Vert _{p-\varepsilon }^{1-\varepsilon }& \leqslant \bigl(\Vert vD\eta \Vert _{p-\varepsilon }+\Vert \eta Dv \Vert _{p-\varepsilon } \bigr)^{1-\varepsilon } \\ &\leqslant \biggl(\frac{C}{R}\Vert v \Vert _{p-\varepsilon }+\Vert Dv \Vert _{p-\varepsilon } \biggr)^{1-\varepsilon } \\ &\leqslant \bigl(CC_{P}^{\frac{1}{p-\varepsilon }}\Vert Dv \Vert _{p-\varepsilon }+ \Vert Dv \Vert _{p-\varepsilon } \bigr)^{1-\varepsilon } \\ &< \bigl(1+C C_{P}^{\frac{1}{p-\varepsilon }} \bigr)\Vert Dv \Vert _{p-\varepsilon }^{1- \varepsilon }. \end{aligned}$$

In view of (3.2) and (3.3), we have

3.4 $$ \Vert h \Vert _{\frac{p-\varepsilon }{1-\varepsilon }}\leqslant C_{1} \varepsilon \Vert Dv \Vert _{p-\varepsilon }^{1-\varepsilon }, $$

where $C_{1}=C_{r}(\Omega ,N)(1+C C_{P}^{\frac{1}{p-\varepsilon }})$.

In particular, combining (3.1) and (3.2), we find

$$\begin{aligned} \Vert D\phi \Vert _{\frac{p-\varepsilon }{1-\varepsilon }} &= \bigl\Vert \bigl\vert D(\eta v) \bigr\vert ^{-\varepsilon }D(\eta v)-h \bigr\Vert _{\frac{p-\varepsilon }{1-\varepsilon }} \\ &\leqslant \bigl\Vert \bigl\vert D(\eta v) \bigr\vert ^{-\varepsilon }D( \eta v) \bigr\Vert _{\frac{p-\varepsilon }{1-\varepsilon }}+ \Vert h \Vert _{\frac{p-\varepsilon }{1-\varepsilon }} \\ &\leqslant \bigl\Vert D (\eta v) \bigr\Vert _{p-\varepsilon }^{1-\varepsilon }+C_{r}( \Omega ,N)\varepsilon \bigl\Vert D (\eta v) \bigr\Vert _{p-\varepsilon }^{1-\varepsilon } \\ &\leqslant \bigl(1+C_{r}(\Omega ,N)\varepsilon \bigr) \bigl\Vert D (\eta v) \bigr\Vert _{p-\varepsilon }^{1-\varepsilon }. \end{aligned}$$

Substituting (3.3) into this estimate, we have

3.5 $$ \Vert D\phi \Vert _{\frac{p-\varepsilon }{1-\varepsilon }} \leqslant C_{2}\Vert Dv \Vert _{p-\varepsilon }^{1-\varepsilon }, $$

where $C_{2}=(1+C_{r}(\Omega ,N)\varepsilon )(1+C C_{P}^{\frac{1}{p- \varepsilon }})$.

Since it is hard to estimate $\vert D(\eta v) \vert ^{-\varepsilon }D(\eta v)$ directly, we set

$$ E(\eta ,v)= \bigl\vert D(\eta v) \bigr\vert ^{-\varepsilon }D( \eta v)-\vert \eta Dv \vert ^{-\varepsilon }\eta Dv, $$

which by Lemma 2 yields

$$ \bigl\vert E(\eta ,v) \bigr\vert \leqslant 2^{\varepsilon} \frac{1+\varepsilon }{1-\varepsilon }\vert vD \eta \vert ^{1-\varepsilon }. $$

Joining $E(\eta ,v)$ with (3.1), we arrive at

$$\begin{aligned} D\phi =E(\eta ,v)+\vert \eta Dv \vert ^{-\varepsilon }\eta Dv-h. \end{aligned}$$

Inserting *Dϕ* into equality (1.2), we get

3.6 $$\begin{aligned} & \int_{B_{R}(x_{0})} A(x, u,Du)\cdot \vert \eta Dv \vert ^{-\varepsilon } \eta Dv \,dx \\ &\quad = \int_{B_{R}(x_{0})} A(x, u,Du)\cdot h\,dx- \int_{B_{R}(x_{0})} A(x, u,Du) \cdot E(\eta ,v)\,dx+ \int_{B_{R}(x_{0})} f(x) \phi \,dx. \end{aligned}$$

In order to use (H2), we need to transform the left-hand side of (3.6) as follows:

$$\begin{aligned} & \int_{B_{R}(x_{0})} A(x, u,Du)\cdot \vert \eta Dv \vert ^{-\varepsilon } \eta Dv \,dx \\ &\quad = \int_{B_{R}(x_{0})} \bigl(A(x, u,Du)-A(x,u,p_{0})+A(x,u,p_{0}) \bigr)\cdot \vert \eta Dv \vert ^{-\varepsilon }\eta Dv\,dx \\ &\quad = \int_{B_{R}(x_{0})} \bigl(A(x, u,Du)-A(x,u, p_{0}) \bigr) \cdot \vert \eta Dv \vert ^{- \varepsilon }\eta Dv\,dx \\ &\quad \quad {}+ \int_{B_{R}(x_{0})} A(x, u,p_{0})\cdot \vert \eta Dv \vert ^{-\varepsilon }\eta Dv\,dx. \end{aligned}$$

Combining this equality with (3.6), we find

3.7 $$\begin{aligned} & \int_{B_{R}(x_{0})} \bigl(A(x, u,Du)-A(x,u, p_{0}) \bigr) \cdot \vert \eta Dv \vert ^{- \varepsilon }\eta Dv\,dx \\ &\quad =- \int_{B_{R}(x_{0})} A(x, u,p_{0}) \cdot \vert \eta Dv \vert ^{-\varepsilon }\eta Dv\,dx+ \int_{B_{R}(x_{0})}A(x,u,Du)\cdot h\,dx \\ &\quad \quad {}- \int_{B_{R}(x_{0})} A(x, u,Du)\cdot E(\eta ,v)\,dx + \int_{B_{R}(x_{0})} f(x)\phi \,dx \\ &\quad \leqslant I_{1}+I_{2}+I_{3}+I_{4}, \end{aligned}$$

where

$$\begin{aligned} &I_{1}= \biggl\vert -\int_{B_{R}(x_{0})} A(x, u,p_{0})\cdot \vert \eta Dv\vert ^{-\varepsilon}\eta Dv\,dx \biggr\vert ; \\ &I_{2}= \biggl\vert \int_{B_{R}(x_{0})}A(x,u,Du)\cdot h\,dx \biggr\vert ; \\ &I_{3}= \biggl\vert - \int_{B_{R}(x_{0})} A(x, u,Du)\cdot E(\eta ,v)\,dx \biggr\vert ; \\ &I_{4}= \biggl\vert \int_{B_{R}(x_{0})} f(x)\phi \,dx \biggr\vert . \end{aligned}$$

Consequently, we shall derive estimate for each term of (3.7) so as to establish a reverse Hölder inequality for $\vert Du-p_{0} \vert ^{p-\varepsilon }$.

In the case of the term on the left-hand side of (3.7), we want to derive an estimate from below in terms of $\int_{B_{R/2}(x_{0})}\vert Du-p_{0} \vert ^{p-\varepsilon }\,dx$. For this purpose, we need the inequality

$$ \bigl(A(x,u,\zeta )-A(x,u,\xi ) \bigr)\cdot (\zeta -\xi )\geqslant \alpha \bigl(1+\vert \zeta \vert ^{2}+\vert \xi \vert ^{2} \bigr)^{\frac{p-2}{2}}\vert \zeta -\xi \vert ^{2}, $$

which can be deduced from (H2) immediately.

Then we infer that

3.8 $$\begin{aligned} & \int_{B_{R}(x_{0})} \bigl(A(x, u,Du)-A(x,u, p_{0}) \bigr) \cdot \vert \eta Dv \vert ^{- \varepsilon }\eta Dv\,dx \\ &\quad = \int_{B_{R}(x_{0})} \vert \eta Dv \vert ^{-\varepsilon }\eta \bigl(A(x, u,Du)-A(x,u, p_{0}) \bigr)\cdot (Du-p_{0})\,dx \\ &\quad \geqslant \alpha \int_{B_{R}(x_{0})}\vert \eta Dv \vert ^{-\varepsilon }\eta \bigl(1+ \vert Du \vert ^{2}+\vert p _{0} \vert ^{2} \bigr)^{\frac{p-2}{2}} \vert Du-p_{0}\vert ^{2}\,dx \\ &\quad \geqslant \alpha \int_{B_{R/2}(x_{0})}\vert Du-p_{0} \vert ^{2-\varepsilon } \biggl(1+\frac{\vert Du-p_{0} \vert ^{2}}{2} \biggr)^{\frac{p-2}{2}}\,dx \\ &\quad \geqslant 2^{\frac{2-p}{2}}\alpha \int_{B_{R/2}(x_{0})}\vert Du-p_{0} \vert ^{p- \varepsilon } \,dx. \end{aligned}$$

Using (H1) and Young’s inequality with exponents $\frac{p-\varepsilon }{1-\varepsilon }$ and $\frac{p-\varepsilon }{p-1}$, we find that, for $\varepsilon_{1}>0$,

3.9 $$\begin{aligned} I_{1}&\leqslant \int_{B_{R}(x_{0})} \bigl\vert A(x, u,p_{0}) \bigr\vert \vert Dv \vert ^{1-\varepsilon }\,dx \\ & \leqslant \beta \bigl(1+\vert p_{0} \vert ^{2} \bigr)^{\frac{p-1}{2}} \int_{B_{R}(x_{0})}\vert Du-p_{0} \vert ^{1-\varepsilon } \,dx \\ & \leqslant \beta \bigl(1+\vert p_{0} \vert ^{2} \bigr)^{\frac{p-1}{2}} \int_{B_{R}(x_{0})} \bigl( \varepsilon_{1}\vert Du-p_{0} \vert ^{p-\varepsilon }+\varepsilon_{1}^{-\frac{1- \varepsilon }{p-1}}1^{\frac{p-\varepsilon }{p-1}} \bigr)\,dx \\ & \leqslant \beta \bigl(1+\vert p_{0} \vert ^{2} \bigr)^{\frac{p-1}{2}}\varepsilon_{1} \int_{B_{R}(x_{0})}\vert Du-p_{0} \vert ^{p-\varepsilon } \,dx \\ &\quad {}+\beta \bigl(1+\vert p_{0} \vert ^{2} \bigr)^{\frac{p-1}{2}}\varepsilon_{1}^{-\frac{1- \varepsilon }{p-1}} \int_{B_{R}(x_{0})}\,dx. \end{aligned}$$

By (H1) we have

$$\begin{aligned} I_{2} & \leqslant \int_{B_{R}(x_{0})} \bigl\vert A(x,u,Du) \bigr\vert \vert h \vert \,dx \\ &\leqslant \beta \int_{B_{R}(x_{0})} \bigl(1+\vert Du \vert ^{2} \bigr)^{\frac{p-1}{2}}\vert h \vert \,dx \\ &\leqslant \beta \int_{B_{R}(x_{0})} \bigl(2+\vert Du-p_{0}+p_{0} \vert ^{2} \bigr)^{ \frac{p-1}{2}}\vert h \vert \,dx \\ &\leqslant \beta \int_{B_{R}(x_{0})} \bigl(2 \bigl(1+\vert p_{0} \vert ^{2} \bigr)+2\vert Du-p_{0} \vert ^{2} \bigr)^{ \frac{p-1}{2}}\vert h \vert \,dx \\ &\leqslant 2^{p-1}\beta \int_{B_{R}(x_{0})}\vert Du-p_{0} \vert ^{p-1} \vert h \vert \,dx+2^{p-1} \beta \bigl(1+\vert p_{0} \vert ^{2} \bigr)^{\frac{p-1}{2}} \int_{B_{R}(x_{0})}\vert h \vert \,dx. \end{aligned}$$

Applying both Hölder’s inequality and Young’s inequality with exponents $\frac{p-\varepsilon }{p-1}$ and $\frac{p-\varepsilon }{1- \varepsilon }$, by (3.4) we further have that, for $\varepsilon_{2}>0$,

3.10 $$\begin{aligned} I_{2}&\leqslant 2^{p-1}\beta \biggl( \int_{B_{R}(x_{0})}\vert Du-p_{0} \vert ^{p-\varepsilon } \,dx \biggr)^{\frac{p-1}{p-\varepsilon }} \biggl( \int_{B_{R}(x_{0})}\vert h \vert ^{\frac{p- \varepsilon }{1-\varepsilon }}\,dx \biggr)^{\frac{1-\varepsilon }{p-\varepsilon }} \\ &\quad +2^{p-1}\beta \bigl(1+\vert p_{0} \vert ^{2} \bigr)^{\frac{p-1}{2}} \biggl( \int_{B_{R}(x _{0})}1^{p-\varepsilon }\,dx \biggr)^{\frac{p-1}{p-\varepsilon }} \biggl( \int_{B_{R}(x_{0})}\vert h \vert ^{\frac{p-\varepsilon }{1-\varepsilon }}\,dx \biggr)^{\frac{1- \varepsilon }{p-\varepsilon }} \\ &\leqslant 2^{p-1}\beta C_{1}\varepsilon \biggl( \int_{B_{R}(x_{0})}\vert Du-p _{0} \vert ^{p-\varepsilon } \,dx \biggr)^{\frac{p-1}{p-\varepsilon }} \biggl( \int_{B_{R}(x_{0})}\vert Du-p_{0} \vert ^{p-\varepsilon } \,dx \biggr)^{\frac{1-\varepsilon }{p-\varepsilon }} \\ &\quad +2^{p-1}\beta \bigl(1+\vert p_{0} \vert ^{2} \bigr)^{\frac{p-1}{2}}C_{1}\varepsilon \biggl( \int_{B_{R}(x_{0})}\vert Du-p_{0} \vert ^{p-\varepsilon }\,dx \biggr)^{\frac{1-\varepsilon }{p-\varepsilon }} \biggl(\int_{B_{R}(x_{0})}\,dx \biggr)^{\frac{p-1}{p-\varepsilon }} \\ &\leqslant 2^{p-1}\beta C_{1}\varepsilon \int_{B_{R}(x_{0})}\vert Du-p _{0} \vert ^{p-\varepsilon } \,dx \\ &\quad +2^{p-1}\beta \bigl(1+\vert p_{0} \vert ^{2} \bigr)^{\frac{p-1}{2}}C_{1}\varepsilon \biggl( \varepsilon_{2} \int_{B_{R}(x_{0})}\vert Du-p_{0} \vert ^{p-\varepsilon } \,dx+ \varepsilon_{2}^{-\frac{1-\varepsilon }{p-1}} \int_{B_{R}(x_{0})}\,dx \biggr) \\ &\leqslant 2^{p-1}\beta C_{1}\varepsilon \int_{B_{R}(x_{0})}\vert Du-p _{0} \vert ^{p-\varepsilon }\,dx+2^{p-1}\beta \bigl(1+ \vert p_{0} \vert ^{2} \bigr)^{\frac{p-1}{2}}C _{1}\varepsilon\varepsilon_{2}^{-\frac{1-\varepsilon }{p-1}} \int_{B_{R}(x_{0})}\,dx \\ &\quad +2^{p-1}\beta \bigl(1+\vert p_{0} \vert ^{2} \bigr)^{\frac{p-1}{2}}C_{1}\varepsilon \varepsilon_{2} \int_{B_{R}(x_{0})}\vert Du-p_{0} \vert ^{p-\varepsilon } \,dx. \end{aligned}$$

Combining (H1) and the estimate of $E(\eta ,v)$, we find that

$$\begin{aligned} I_{3}&\leqslant \int_{B_{R}(x_{0})} \bigl\vert A(x, u,Du) \bigr\vert \bigl\vert E( \eta ,v) \bigr\vert \,dx \\ &\leqslant \int_{B_{R}(x_{0})} \beta \bigl(1+\vert Du \vert ^{2} \bigr)^{\frac{p-1}{2}}2^{ \varepsilon} \frac{1+\varepsilon }{1-\varepsilon }\vert vD \eta \vert ^{1- \varepsilon }\,dx \\ &\leqslant \beta 2^{\varepsilon} \frac{1+\varepsilon }{1-\varepsilon } \biggl(\frac{C}{R} \biggr)^{1-\varepsilon } \int_{B_{R}(x_{0})} \bigl(1+\vert Du \vert ^{2} \bigr)^{ \frac{p-1}{2}}\vert v \vert ^{1-\varepsilon }\,dx. \\ &\leqslant \beta 2^{\varepsilon} \frac{1+\varepsilon }{1-\varepsilon } \biggl(\frac{C}{R}\biggr)^{1-\varepsilon } \int_{B_{R}(x_{0})} \bigl(2 \bigl(1+\vert p_{0} \vert ^{2} \bigr)+2\vert Du-p _{0}\vert ^{2} \bigr)^{\frac{p-1}{2}} \vert v \vert ^{1-\varepsilon } \,dx \\ &\leqslant \beta 2^{\varepsilon} \frac{1+\varepsilon }{1-\varepsilon } \biggl(\frac{C}{R} \biggr)^{1-\varepsilon }2^{p-1} \int_{B_{R}(x_{0})} \bigl( \bigl(1+\vert p_{0} \vert ^{2} \bigr)^{ \frac{p-1}{2}}+\vert Du-p_{0} \vert ^{p-1} \bigr)\vert v \vert ^{1-\varepsilon }\,dx. \end{aligned}$$

Denoting $\beta 2^{\varepsilon} \frac{1+\varepsilon }{1-\varepsilon }( \frac{C}{R})^{1-\varepsilon }2^{p-1}$ by $C_{3}$, we have

3.11 $$ I_{3} \leqslant C_{3} \bigl(1+\vert p_{0} \vert ^{2} \bigr)^{\frac{p-1}{2}}K_{1}+C_{3}K _{2}, $$

where $K_{1}=\int_{B_{R}(x_{0})} \vert v \vert ^{1-\varepsilon }\,dx$ and $K_{2}=\int_{B_{R}(x_{0})}\vert Du-p_{0} \vert ^{p-1}\vert v \vert ^{1-\varepsilon }\,dx$. Let us estimate $K_{1}$ and $K_{2}$. Using Young’s inequality with exponents $\frac{p-\varepsilon }{1-\varepsilon }$ and $\frac{p-\varepsilon }{p-1}$ and Poincaré’s inequality with constant $C_{P}$, we find that, for $\varepsilon_{3}>0$,

3.12 $$\begin{aligned} K_{1} &\leqslant \int_{B_{R}(x_{0})} \bigl(\varepsilon_{3}\vert v \vert ^{p-\varepsilon }+\varepsilon_{3}^{-\frac{1-\varepsilon }{p-1}}1^{\frac{p-\varepsilon }{p-1}} \bigr) \,dx \\ &\leqslant \varepsilon_{3} \int_{B_{R}(x_{0})}\vert v \vert ^{p-\varepsilon }\,dx+ \varepsilon_{3}^{-\frac{1-\varepsilon }{p-1}} \int_{B_{R}(x_{0})}\,dx \\ &\leqslant \varepsilon_{3}C_{P} \int_{B_{R}(x_{0})}\vert Du-p_{0} \vert ^{p- \varepsilon } \,dx+\varepsilon_{3}^{-\frac{1-\varepsilon }{p-1}} \int_{B_{R}(x_{0})}\,dx. \end{aligned}$$

Letting $p'=\frac{n(p-\varepsilon )}{(n+1-\varepsilon )(p-1)}$ and $q'=\frac{n(p-\varepsilon )}{(n-p+1)(1-\varepsilon )}$, we see that $1< p'<\infty $, $1< q'<\infty $, and $\frac{1}{p'}+\frac{1}{q'}=1$. With the aid of Hölder’s inequality, we can estimate

$$\begin{aligned} K_{2} &\leqslant \biggl( \int_{B_{R}(x_{0})}\vert Du-p_{0} \vert ^{(p-1)p'} \,dx \biggr)^{ \frac{1}{p'}} \biggl( \int_{B_{R}(x_{0})}\vert v \vert ^{(1-\varepsilon )q'}\,dx \biggr)^{ \frac{1}{q'}} \\ &\leqslant \biggl( \int_{B_{R}(x_{0})}\vert Du-p_{0} \vert ^{\frac{n(p-\varepsilon )}{n+1- \varepsilon }} \,dx \biggr)^{\frac{(n+1-\varepsilon )(p-1)}{n(p-\varepsilon )}} \biggl( \int_{B_{R}(x_{0})}\vert v \vert ^{\frac{n(p-\varepsilon )}{n-p+1}}\,dx \biggr)^{\frac{(n-p+1)(1- \varepsilon )}{n(p-\varepsilon )}}. \end{aligned}$$

Now we set $p''=\frac{n(p-\varepsilon )}{n+1-\varepsilon }$. Then $\frac{np''}{n-p''}=\frac{n(p-\varepsilon )}{n-p+1}$. Using the Sobolev-Poincaré inequality with constant $C_{s}$, we get

3.13 $$\begin{aligned} K_{2} &\leqslant C_{s}^{1-\varepsilon } \biggl( \int_{B_{R}(x_{0})}\vert Du-p_{0} \vert ^{\frac{n(p- \varepsilon )}{n+1-\varepsilon }} \,dx \biggr)^{\frac{(n+1-\varepsilon )(p-1)}{n(p- \varepsilon )}} \biggl( \int_{B_{R}(x_{0})}\vert Du-p_{0} \vert ^{\frac{n(p-\varepsilon )}{n+1- \varepsilon }} \,dx \biggr)^{\frac{(n+1-\varepsilon )(1-\varepsilon )}{n(p- \varepsilon )}} \\ &\leqslant C_{s}^{1-\varepsilon } \biggl( \int_{B_{R}(x_{0})}\vert Du-p_{0} \vert ^{\frac{n(p- \varepsilon )}{n+1-\varepsilon }} \,dx \biggr)^{\frac{n+1-\varepsilon }{n}}. \end{aligned}$$

Combining (3.11) with (3.12) and (3.13), we obtain the estimate for $I_{3}$:

3.14 $$\begin{aligned} I_{3} &\leqslant C_{3} \bigl(1+\vert p_{0} \vert ^{2} \bigr)^{\frac{p-1}{2}} \varepsilon_{3}C _{P} \int_{B_{R}(x_{0})}\vert Du-p_{0} \vert ^{p-\varepsilon } \,dx \\ &\quad {}+C_{3} \bigl(1+\vert p_{0} \vert ^{2} \bigr)^{\frac{p-1}{2}}\varepsilon_{3}^{-\frac{1- \varepsilon }{p-1}} \int_{B_{R}(x_{0})}\,dx \\ &\quad {}+C_{3} C_{s}^{1-\varepsilon } \biggl( \int_{B_{R}(x_{0})}\vert Du-p_{0} \vert ^{\frac{n(p- \varepsilon )}{n+1-\varepsilon }} \,dx \biggr)^{\frac{n+1-\varepsilon }{n}}. \end{aligned}$$

Finally, we estimate $I_{4}$. Using Hölder’s inequality with exponents $\frac{n(p-\varepsilon )}{n(p-1)+p-\varepsilon }$ and $\frac{n(p-\varepsilon )}{n(1-\varepsilon )-p+\varepsilon }$, we have

$$\begin{aligned} I_{4} & \leqslant \int_{B_{R}(x_{0})} \bigl\vert f(x) \bigr\vert \vert \phi \vert \,dx \\ & \leqslant \biggl( \int_{B_{R}(x_{0})}\vert f \vert ^{\frac{n(p-\varepsilon )}{n(p-1)+p- \varepsilon }}\,dx \biggr)^{\frac{n(p-1)+p-\varepsilon }{n(p-\varepsilon )}} \biggl( \int_{B_{R}(x_{0})}\vert \phi \vert ^{\frac{n(p-\varepsilon )}{n(1-\varepsilon )-p+\varepsilon }}\,dx \biggr)^{\frac{n(1-\varepsilon )-p+\varepsilon }{n(p- \varepsilon )}}. \end{aligned}$$

Setting $p'''=\frac{p-\varepsilon }{1-\varepsilon }$, we have $\frac{np'''}{n-p'''}=\frac{n(p-\varepsilon )}{n(1-\varepsilon )-p+ \varepsilon }$. Notice that $\phi \in W_{0}^{1,\frac{p-\varepsilon }{1- \varepsilon }}(B_{R}(x_{0}))$. Therefore, we can apply the Sobolev-Poincaré inequality to get

$$\begin{aligned} I_{4}\leqslant C_{s} \biggl( \int_{B_{R}(x_{0})}\vert f \vert ^{\frac{n(p-\varepsilon )}{n(p-1)+p- \varepsilon }}\,dx \biggr)^{\frac{n(p-1)+p-\varepsilon }{n(p-\varepsilon )}} \biggl( \int_{B_{R}(x_{0})}\vert D\phi \vert ^{\frac{p-\varepsilon }{1-\varepsilon }}\,dx \biggr)^{\frac{1- \varepsilon }{p-\varepsilon }}. \end{aligned}$$

Combining this with (3.5), with the aid of Young’s inequality, we obtain, for $\varepsilon_{4}>0$,

3.15 $$\begin{aligned} I_{4}&\leqslant C_{s} C_{2} \biggl( \int_{B_{R}(x_{0})}\vert f \vert ^{\frac{n(p- \varepsilon )}{n(p-1)+p-\varepsilon }}\,dx \biggr)^{\frac{n(p-1)+p-\varepsilon }{n(p-\varepsilon )}} \biggl( \int_{B_{R}(x_{0})}\vert Du-p_{0} \vert ^{p-\varepsilon } \,dx \biggr)^{\frac{1- \varepsilon }{p-\varepsilon }} \\ &\leqslant C_{s} C_{2} \biggl(\varepsilon_{4} \int_{B_{R}(x_{0})}\vert Du-p_{0} \vert ^{p- \varepsilon } \,dx+\varepsilon_{4}^{-\frac{1-\varepsilon }{p-1}} \biggl( \int_{B_{R}(x_{0})}\vert f \vert ^{\frac{n(p-\varepsilon )}{n(p-1)+p-\varepsilon }}\,dx \biggr)^{\frac{n(p-1)+p-\varepsilon }{n(p-1)}} \biggr). \end{aligned}$$

Substituting (3.8), (3.9), (3.10), (3.14), and (3.15) into (3.7), we finally have

$$\begin{aligned} & 2^{\frac{2-p}{2}}\alpha \int_{B_{R/2}(x_{0})}\vert Du-p_{0} \vert ^{p-\varepsilon } \,dx \\ &\quad \leqslant \beta \bigl(1+\vert p_{0} \vert ^{2} \bigr)^{\frac{p-1}{2}}\varepsilon_{1} \int_{B_{R}(x_{0})}\vert Du-p_{0} \vert ^{p-\varepsilon } \,dx+2^{p-1}\beta C_{1} \varepsilon \int_{B_{R}(x_{0})}\vert Du-p_{0} \vert ^{p-\varepsilon } \,dx \\ &\quad\quad {}+2^{p-1}\beta \bigl(1+\vert p_{0} \vert ^{2} \bigr)^{\frac{p-1}{2}}C_{1}\varepsilon \varepsilon_{2} \int_{B_{R}(x_{0})}\vert Du-p_{0} \vert ^{p-\varepsilon } \,dx \\ &\quad \quad {}+C_{3} \bigl(1+\vert p_{0} \vert ^{2} \bigr)^{\frac{p-1}{2}}\varepsilon_{3}C_{P} \int_{B_{R}(x_{0})}\vert Du-p_{0} \vert ^{p-\varepsilon } \,dx +C_{s} C_{2} \varepsilon_{4} \int_{B_{R}(x_{0})}\vert Du-p_{0} \vert ^{p-\varepsilon } \,dx \\ &\quad \quad {}+C_{3} C_{s}^{1-\varepsilon } \biggl( \int_{B_{R}(x_{0})}\vert Du-p_{0} \vert ^{\frac{n(p- \varepsilon )}{n+1-\varepsilon }} \,dx \biggr)^{\frac{n+1-\varepsilon }{n}} \\ &\quad \quad {} +C _{s} C_{2} \varepsilon_{4}^{-\frac{1-\varepsilon }{p-1}} \biggl( \int_{B_{R}(x_{0})}\vert f \vert ^{\frac{n(p-\varepsilon )}{n(p-1)+p-\varepsilon }}\,dx \biggr)^{\frac{n(p-1)+p-\varepsilon }{n(p-1)}} \\ &\quad\quad {} +\beta \bigl(1+\vert p_{0} \vert ^{2} \bigr)^{\frac{p-1}{2}}\varepsilon_{1}^{-\frac{1- \varepsilon }{p-1}} \int_{B_{R}(x_{0})}\,dx+2^{p-1}\beta \bigl(1+\vert p_{0} \vert ^{2} \bigr)^{ \frac{p-1}{2}}C_{1} \varepsilon \varepsilon_{2}^{-\frac{1-\varepsilon }{p-1}} \int_{B_{R}(x_{0})}\,dx \\ &\quad \quad {}+C_{3} \bigl(1+\vert p_{0} \vert ^{2} \bigr)^{\frac{p-1}{2}}\varepsilon_{3}^{-\frac{1- \varepsilon }{p-1}} \int_{B_{R}(x_{0})}\,dx. \end{aligned}$$

Rearranging this inequality, we have

$$\begin{aligned} & \int_{B_{R/2}(x_{0})}\vert Du-p_{0} \vert ^{p-\varepsilon } \,dx \\ &\quad \leqslant \theta \int_{B_{R}(x_{0})}\vert Du-p_{0} \vert ^{p-\varepsilon } \,dx +\frac{1}{ \alpha }2^{\frac{p-2}{2}}C_{3} C_{s}^{1-\varepsilon } \biggl( \int_{B_{R}(x _{0})}\vert Du-p_{0} \vert ^{\tau } \,dx \biggr)^{\frac{p-\varepsilon }{\tau }} \\ &\quad \quad {}+\frac{1}{\alpha }2^{\frac{p-2}{2}}C_{s} C_{2} \varepsilon _{4}^{-\frac{1-\varepsilon }{p-1}} \biggl( \int_{B_{R}(x_{0})}\vert f \vert ^{\frac{n(p- \varepsilon )}{n(p-1)+p-\varepsilon }}\,dx \biggr)^{\frac{n(p-1)+p-\varepsilon }{n(p-1)}} +C_{4} \int_{B_{R}(x_{0})}\,dx, \end{aligned}$$

where $\theta =\frac{1}{\alpha }2^{\frac{p-2}{2}}(\beta (1+\vert p_{0} \vert ^{2})^{ \frac{p-1}{2}}\varepsilon_{1}+2^{p-1}\beta C_{1}\varepsilon +2^{p-1} \beta (1+\vert p_{0} \vert ^{2})^{\frac{p-1}{2}}C_{1}\varepsilon \varepsilon_{2}+C _{3} (1+\vert p_{0} \vert ^{2})^{\frac{p-1}{2}} \varepsilon_{3}C_{P}+C_{s} C_{2} \varepsilon_{4})$, $\tau =\frac{n(p-\varepsilon )}{n+1-\varepsilon }$, and $C_{4}=\frac{1}{\alpha }2^{\frac{p-2}{2}}(\beta (1+\vert p_{0} \vert ^{2})^{ \frac{p-1}{2}}\varepsilon_{1}^{-\frac{1-\varepsilon }{p-1}}+2^{p-1} \beta (1+\vert p_{0} \vert ^{2})^{\frac{p-1}{2}}C_{1}\varepsilon \varepsilon_{2} ^{-\frac{1-\varepsilon }{p-1}}+C_{3}(1+\vert p_{0} \vert ^{2})^{\frac{p-1}{2}} \varepsilon_{3}^{-\frac{1-\varepsilon }{p-1}})$.

By (H4) we get $\frac{n(p-\varepsilon )}{n(p-1)+p-\varepsilon }< \frac{nq}{n(p-1)+q}$ and $\int_{B_{R}(x_{0})}\vert f \vert ^{\frac{n(p-\varepsilon )}{n(p-1)+p-\varepsilon }}\,dx< M$.

Moreover, we have $(\int_{B_{R}(x_{0})}\vert f \vert ^{\frac{n(p-\varepsilon )}{n(p-1)+p- \varepsilon }}\,dx)^{\frac{n(p-1)+p-\varepsilon }{n(p-1)}}\leqslant M ^{\frac{p-\varepsilon }{n(p-1)}}\int_{B_{R}(x_{0})}\vert f \vert ^{\frac{n(p- \varepsilon )}{n(p-1)+p-\varepsilon }}\,dx$.

Setting $C_{5}=\max \{\frac{1}{\alpha }2^{\frac{p-2}{2}}C_{s} C_{2} \varepsilon_{4}^{-\frac{1-\varepsilon }{p-1}}M^{ \frac{p-\varepsilon }{n(p-1)}},C_{4}\}$, we have

$$\begin{aligned} & \int_{B_{R/2}(x_{0})}\vert Du-p_{0} \vert ^{p-\varepsilon } \,dx \\ &\quad \leqslant \theta \int_{B_{R}(x_{0})}\vert Du-p_{0} \vert ^{p-\varepsilon } \,dx +\frac{1}{ \alpha }2^{\frac{p-2}{2}}C_{3} C_{s}^{1-\varepsilon } \biggl( \int_{B_{R}(x _{0})}\vert Du-p_{0} \vert ^{\tau } \,dx \biggr)^{\frac{p-\varepsilon }{\tau }} \\ &\quad\quad {} +C_{5} \biggl( \int_{B_{R}(x_{0})}\vert f \vert ^{\frac{n}{n(p-1)+p-\varepsilon }(p-\varepsilon )}\,dx + \int_{B_{R}(x_{0})}\,dx \biggr) \\ &\quad \leqslant \theta \int_{B_{R}(x_{0})}\vert Du-p_{0} \vert ^{p-\varepsilon } \,dx +\frac{1}{ \alpha }2^{\frac{p-2}{2}}C_{3} C_{s}^{1-\varepsilon } \biggl( \int_{B_{R}(x _{0})}\vert Du-p_{0} \vert ^{\tau } \,dx \biggr)^{\frac{p-\varepsilon }{\tau }} \\ &\quad \quad {}+C_{5} \int_{B_{R}(x_{0})} \bigl(\vert f \vert ^{\frac{n}{n(p-1)+p-\varepsilon }}+1 \bigr)^{p-\varepsilon }\,dx. \end{aligned}$$

Dividing both sides by $\vert B_{R}(x_{0}) \vert =\alpha_{n} R^{n}$ yields

3.16 $$\begin{aligned}& \fint_{B_{R/2}(x_{0})}\vert Du-p_{0} \vert ^{p-\varepsilon } \,dx \\& \quad \leqslant 2^{n}\theta - \int_{B_{R}(x_{0})}\vert Du-p_{0} \vert ^{p-\varepsilon } \,dx +C_{6} \biggl( \fint_{B_{R}(x_{0})}\vert Du-p_{0} \vert ^{\tau } \,dx \biggr)^{\frac{p-\varepsilon }{ \tau }} \\& \quad \quad {}+2^{n}C_{5} \fint_{B_{R}(x_{0})} \bigl(\vert f \vert ^{\frac{n}{n(p-1)+p-\varepsilon }}+1 \bigr)^{p- \varepsilon }\,dx, \end{aligned}$$

where $C_{6}=\frac{2^{n}}{\alpha }2^{\frac{p-2}{2}}C_{3} C_{s}^{1- \varepsilon }(\alpha_{n}R^{n})^{\frac{p-\tau -\varepsilon }{\tau }}$.

Taking $\varepsilon ,\varepsilon_{1},\varepsilon_{2},\varepsilon_{3}, \varepsilon_{4}$ sufficiently small such that $2^{n}\theta <1$ and $\tau >1$, we obtain the reverse Hölder inequality for $\vert Du-p_{0} \vert ^{p- \varepsilon }$. Accordingly, by Lemma 2 we can derive that *u* belongs to $W^{1,r'}_{\mathrm{loc}}(\Omega )$ with $r'>r$. Since $f\in L_{\mathrm{loc}}^{ \frac{nq}{n(p-1)+q}}(\Omega )$, $q>p$, reasoning as before, we get a new estimate analogous to (3.16) with exponents $r'$ and $\tau '$ in place of *r*, that is, $p-\varepsilon $ and *τ*, respectively:

$$\begin{aligned}& \fint_{B_{R/2}(x_{0})}\vert Du-p_{0} \vert ^{r'}\,dx \\& \quad \leqslant 2^{n}\theta ' \fint_{B_{R}(x_{0})}\vert Du-p_{0} \vert ^{r'}\,dx +C_{6}' \biggl( \fint_{B_{R}(x_{0})}\vert Du-p_{0} \vert ^{\tau '}\,dx \biggr)^{\frac{r'}{\tau '}} \\& \quad\quad {} +2^{n}C_{5}' \fint_{B_{R}(x_{0})} \bigl(\vert f \vert ^{\frac{n}{n(p-1)+r'}}+1 \bigr)^{r'}\,dx. \end{aligned}$$

Therefore, we get $u\in W^{1,r''}_{\mathrm{loc}}(\Omega )$ with $r''>r'$. Repeating this process, we can improve the degree of integrability of *Du* again and again. Thus, it is clear that $u\in W^{1,t}_{\mathrm{loc}}( \Omega )$ with any $t\in (r_{1},r_{2})$.

This completes the proof of Theorem 1. □

### Proof of Theorem 2

#### Proof

The aim of Theorem 2 is to prove that very weak solutions to systems (1.1) are not only weak solutions to (1.1) but also the optimal Hölder continuity. In fact, under the assumptions of Theorem 1, we get that $u\in W_{\mathrm{loc}}^{1,r}(\Omega )$ with $r_{1}< r< p$ are weak solutions $u\in W_{\mathrm{loc}}^{1,p}(\Omega )$ to systems (1.1) by Corollary 1. Then we can safely infer $u\in C^{1,\gamma }(\Omega_{0})$ based on A-harmonic approximation technique. The proving method is standard, so we omit the process of derivation in this paper. For more details, we refer the reader to Theorem 1.1 of [14] and the related literature. So the proof of Theorem 2 is complete. □
